# Perspectives of parents and nurses on the content validity of the Family Empowerment Scale for parents of children with a chronic condition: A mixed‐methods study

**DOI:** 10.1111/cch.12629

**Published:** 2018-12-12

**Authors:** Elisabeth W. Segers, Agnes van den Hoogen, Irene C. van Eerden, Thora Hafsteinsdóttir, Marjolijn Ketelaar

**Affiliations:** ^1^ Department of Children, Wilhelmina Children's Hospital University Medical Center Utrecht Utrecht The Netherlands; ^2^ Department of Neonatology, Wilhelmina Children's Hospital University Medical Centre Utrecht Utrecht The Netherlands; ^3^ Princess Máxima Center for Pediatric Oncology Utrecht The Netherlands; ^4^ Department of Nursing Science, Julius Center for Health Science and Primary Care University Medical Center Utrecht Utrecht The Netherlands; ^5^ Center of Excellence for Rehabilitation Medicine, Brain Center Rudolf Magnus University Medical Center Utrecht and De Hoogstraat Rehabilitation Utrecht The Netherlands

**Keywords:** children, chronic conditions, content validity, empowerment, Family Empowerment Scale, parents

## Abstract

**Background:**

Insight into parental empowerment is important to understanding the impact of health care policy and to supporting and strengthening parents in the care of their child. The Family Empowerment Scale (FES) is a valid 24‐item instrument that measures parental empowerment. It was originally developed for parents of children with emotional disabilities. It has been translated from English into Dutch.

Before using the translated FES in another context, the aim of this study was to assess the content validity of the Dutch FES in the context of children with a chronic condition in a children's hospital, according to parents and nurses.

**Method:**

This content validity study has a convergent, mixed‐methods design. The content validity index was used to examine the relevance, according to 22 parents and 12 nurses quantitatively, on a scale and item level. The qualitative part assessed the comprehensiveness and comprehension of the FES through cognitive interviewing with eight parents and four nurses. The results of both analyses were converged to determine content validity.

**Results:**

The scale‐content validity index was 0.88; three items scored < 0.78 on the item level. For 10 (of 24) items, issues were noticed about the tone and clarity of wording. Participants considered the FES to be not only an instrument of research but also an instrument that could be used to give insight into the personal degree of parental empowerment.

**Conclusion:**

The content validity of the Dutch FES for parents of children with a chronic condition can be considered sufficient. Resolving some minor translation issues in some of the items is advised. The FES can be used in further research to examine the value of the FES in health care services, aiming to support the needs of parents and to increase their empowerment.

**Key messages**

- The Dutch Family Empowerment Scale (FES) is a 24‐item rating scale that provides insight into parents' sense of their own empowerment at one particular point in time. It consists of two domains: family and service systems. The FES covers three expressions of empowerment: attitudes, knowledge, and behaviours. The FES has been translated into various languages, including Dutch.
- This study shows sufficient content validity of the Dutch FES for parents of children with a chronic condition in a hospital setting. The items and questionnaire were considered relevant and comprehensive by both parents and nurses
- The FES has been primarily used to evaluate interventions in research, but it has the potential to be used to assess parental empowerment with the aim of providing individualized support.

## 1 INTRODUCTION

In past few decades, there has been growing attention paid to the empowerment of patients' families, especially to the parents of a child with a chronic condition, as they are the primary caregivers of their child (Coffey, 2006; Holmström & Röling, 2010; Smith, Swallow, & Coyne, 2015). Parents of a child with a chronic condition often provide complex care and treatment and manage their child's conditions (Gannoni & Shute, 2010; Gibson, 1995; Smith et al., 2015). They face challenges that are different from those of families with healthy children, often including increased worries and distress (Cashin, Small, & Solberg, 2008; Coffey, 2006; Swallow, Lambert, Santacroce, & Macfadyen, 2011). It is important for parents to be able to face these challenges and to be empowered to participate in decisions and the supervision of the care of their child (Gibson, 1995; Hallström & Elander, 2007; Payrovee, Kashaninia, Alireza Mahdaviani, & Rezasoltani, 2014; Vuorenmaa, Halme, Astedt‐Kurli, Kaunonen, & Perälä, 2014).

Empowerment is considered to be an important concept in strengthening parents' position in health care (Barlow & Ellard, 2004; Hook, 2006). Although empowerment is described in different ways, it can be defined as a sense of power that gives the ability to influence people, organizations, and environments, and it also gives one control over one's life (Fumagalli, Radaelli, Lettieri, & Masella, 2015; Koren, DeChillo, & Friesen, 1992; Vuorenmaa et al., 2014). Increased parental empowerment has a positive impact on well‐being, self‐efficacy, and levels of stress, and it is associated with an improved ability of parents to make adequate choices regarding their children's treatment (Koren et al., 1992; Vuorenmaa et al., 2014).

Insight into parental empowerment is important for several reasons (McAllister, Dunn, Payne, Davies, & Todd, 2012). It provides the opportunity to understand whether implemented care interventions effectively contribute to supporting and strengthening parents. Furthermore, it provides insight into the perceived empowerment of individual parents, so that customized support for parents may be provided.

The Family Empowerment Scale (FES) was developed in 1992 by Koren et al. as a brief, self‐administered, 34‐item measurement scale. The original version of the FES was developed for parents whose children had emotional disabilities (Koren et al., 1992). The FES has been translated into many languages, including Finnish, Hebrew, Japanese, Spanish, and, recently, Dutch (Florian & Elad, 1998; Kageyama & Nakamura, 2016; Ketelaar et al., 2010; Martínez, Pérez, Ramírez, Canino, & Rand, 2009; Vuorenmaa et al., 2014). The FES is increasingly used and validated in different populations, including in children with diabetes and the parents of small children (Florian & Elad, 1998; Vuorenmaa et al., 2014)

Although the FES is a valid and reliable instrument for use in different populations and cultural environments than it was originally developed for, it requires re‐examination of its psychometric properties (Guillemin, 1993; Singh, Curtis, & Ellis, 1995). Until now, the FES has neither been used nor validated in a population of parents of children with a chronic condition. When considering the psychometric properties, one of the first steps is to assess the content validity. If the content validity is adequate, evaluation of other measurement properties is useful (Terwee et al., 2007). Content validity is defined as the degree to which the content of a questionnaire is an adequate reflection of the construct to be measured; it should be assessed by experts who can make a judgement about the relevance and the comprehensiveness of the items (Mokkink et al., 2010).

The aim of this study is to assess the content validity of the Dutch FES in the context of children with a chronic condition in a children's hospital setting, according to their parents and nurses.

## 2 METHODS

### 2.1 Family Empowerment Scale

The original FES consists of 34 items in three domains, family (12 items), service system (12 items), and involvement in community (10 items), and refers to three expressions of empowerment, attitudes, knowledge, and behaviours (Koren et al., 1992). Different studies have demonstrated that the psychometric properties of the FES are robust in both the original and translated versions (Koren et al., 1992; Singh et al., 1995; Itzhaky & Schwartz, 2001; Vuorenmaa et al., 2014).

In an earlier stage, the FES has been translated in Dutch and discussed with parents and health care providers. Based on these discussions, it was decided, in close coordination with the developers of the FES, not to translate the third domain. The items in this third domain, Involvement in the community, were felt to be too culturally specific and not applicable to Dutch context. The Dutch translation of the FES therefore consists of 24 items. After this stage, the Dutch translation of the FES was back‐translated into English by an independent translator. The Dutch translators and the authors of the original FES compared the backward translation with the original version and confirmed the Dutch translation.

### 2.2 Design

This content validity study has a convergent, mixed‐methods design. To provide reliable and complete outcomes, the two concepts of content validity (relevance and comprehensiveness) were examined separately in the same population. Data from both parts were collected during a similar timeframe and initially analysed separately before being compiled into a final analysis (Zhang & Creswell, 2013).

The quantitative part was a cross‐sectional observational study that assessed the relevance of the items in the Dutch FES on a 4‐point scale. A short explanation about the definition of empowerment was added to the FES, as a guiding principle for assessing relevance (Polit, Beck, & Owen, 2007). In the qualitative part, the comprehensibility of the items and the comprehensiveness of the questionnaire were assessed through cognitive interviewing (Beatty & Willis, 2007; Patrick et al., 2011).

The Medical Research Ethics Committee of the UMC Utrecht confirmed that the Dutch Medical Research Involving Human Subjects Act (WMO) did not apply to this study, Protocol number 17‐035‐C. After giving information and an explanation of the study, verbal and written consent of the participants was obtained.

### 2.3 Population and sample

The population for both parts of this study consisted of the parents of children with a chronic condition who were receiving treatment in an academic children's hospital and their nurses and nurse specialists, as they are both considered experts in parental empowerment (Mokkink et al., 2010). The study was conducted in an academic children's hospital in the Netherlands and took 6 months, from January 2017 to June 2017.

Included in the study were the parents of a child with a chronic condition. A chronic condition is defined by Mokkink, van der Lee, Grootenhuis, Offringa, and Heymans (2008) as “an illness that occurs from the age of 0 to 18 years; the diagnosis is based on scientific medical knowledge and can be established using reproducible and valid methods and instruments according to professionals; it is not (yet) curable or, for mental health conditions, is highly resistant to treatment and has been present for longer than three months, or it has a high probability of lasting longer than three months, or it has occurred three times or more during the past year with a high likelihood of recurrence.” Nurses and nurse specialists, experienced in the care of children with a chronic condition as defined by Mokkink et al., were included when they had at least 1 year of work experience. Participants had to be able to speak, write, and read Dutch.

To provide rich data, the parents of children with different chronic diseases, different gender, age, education level, and duration of child's illness and nurses with experience in different chronic illness were selected by purposeful sampling (Beatty & Willis, 2007). All participants were selected by the researcher. The electronic patient file was searched, and clinicians were asked to find eligible parents who came to the hospital for an appointment or treatment. For the quantitative part of the study, 34 parents and 12 nurses were approached. From this sample, four nurses and eight parents were purposefully selected and approached by the researcher for the qualitative part, based on variation in outcomes of the quantitative data.

### 2.4 Procedure

Eligible parents were invited to participate in this study by the child's clinician, by a nurse, or by the researcher. Parents of children visiting the outpatient clinics were asked by their attending clinician. Nurses were asked by the researcher.

After signing informed consent, participants received a questionnaire focusing on the relevance of the FES, and demographic characteristics were obtained. Parents received the materials either by mail, which included a reply envelope, or in person when present at the hospital; nurses received the materials in person. When no response was received after 2 weeks, participants were reminded by a telephone call.

For participants in the qualitative part of the study, an appointment was made for an interview. The interviews were audio recorded with permission of the participants.

### 2.5 Outcomes

The relevance of the Dutch FES was expressed in content validity index on an item level (I‐CVI) and scale level (S‐CVI). The I‐CVI is the number of experts giving an item of the Dutch FES a score of either 3 or 4 on the 4‐point relevance scale, divided by the total number of experts. Items with an I‐CVI ≥ 0.78 are judged to be relevant, taking into account the risk of chance agreement. The CVI on the scale level was calculated by averaging the I‐CVI values. A score ≥0.90 is considered good; 0.80 is considered sufficient and is the lower limit of acceptability for an S‐CVI (Polit et al., 2007).

The comprehensiveness of the questionnaire and the comprehensibility of the items were evaluated through cognitive interviewing, assessing participants' understanding and interpretation of the questionnaire (Beatty & Willis, 2007; Patrick et al., 2011). To get insight into participants' decisions regarding what constituted appropriate responses, participants were encouraged to think aloud when giving their interpretation of the Dutch FES items. Subsequently, in order to inquire about aspects of the concept that had not been covered, as well as the complexity of the questionnaire, for which an interview guide was used, the researcher asked probing questions to establish that the item was understood correctly (Beatty & Willis, 2007; Patrick et al., 2011). Interpretation of comprehensibility and comprehensiveness was based on comments raised by parents and their interpretation of items and the whole questionnaire.

### 2.6 Analysis

#### 2.6.1 Quantitative part

The relevance of the Dutch FES was expressed in the CVI (Lynn, 1986; Polit et al., 2007). For each item, the I‐CVI, and for the total scale, the S‐CVI was calculated (Polit et al., 2007).

If an item was not completed by all of the experts, the I‐CVI was calculated by dividing the number of experts giving a 3 or 4 rating by the total number of experts who rated this item. IBM SPSS version 22 (Armonk, New York, USA) was used.

#### 2.6.2 Qualitative part

To give insight into the comprehensiveness and comprehensibility of the FES, analysis of the interviews was carried out following the method described by Knafl et al. (2007). This method takes the individual item as a basis for the analysis and distinguishes between interpretations and issues that participants made regarding the items.

Data were transcribed verbatim. The interpretations of all of the participants were categorized per item in a scheme, along with their comments, in order to facilitate a comparison of the participants' interpretations. To standardize and increase the quality of the analysis, two researchers reviewed the first five interviews for half of the items.

A summary was made of the interpretations and findings for each item. Two researchers analysed this summary to determine what the major interpretations and comments were. The comments were analysed to create codes, according to Knafl (Knafl et al., 2007; Patrick et al., 2011). Consensus was reached by discussing and reviewing the analyses. An overview was made, which evaluated the comprehensibility of the items and the comprehensiveness and complexity of the questionnaire.

#### 2.6.3 Converging quantitative and qualitative parts

All outcomes of the quantitative and qualitative data were combined to provide insight into the content validity of the questionnaire and to give the joint recommendations of the two researchers (Knafl et al., 2007).

Four categories of items were formed: (a) Relevant and comprehensible items had a CVI score ≥ 0.78, a consistent interpretation or no more than one interpretation that varied substantially from all others, and minimal problems, as noted by three or less participants. They were recommended to be retained. (b) Relevant items that were incomprehensible had a CVI score ≥ 0.78 and had important and substantially varied interpretations or comments noted by three or more participants concerning the comprehensibility of the questionnaire. These items were recommended to be modified and could be retained after modification. (c) Irrelevant but comprehensible items had CVI < 0.78 and a consistent interpretation, as described as above. Outcomes of the qualitative part of the irrelevant items were reevaluated based on participants' comments, (e.g., incomprehensibility by wording or tone), in order to make specific recommendations to modify or to retain them. (d) Irrelevant and incomprehensible items had a CVI score < 0.78 and substantially varied interpretations or comments concerning the comprehensibility of the questionnaire. They were recommended to be deleted.

Finally, two independent researchers reviewed and assessed the analysis process, and discussed the process and recommendations with the developers of the original questionnaire to make final decisions about adjustments.

## 3 RESULTS

### 3.1 Participants

For the quantitative part of the study, 34 parents were invited, and 22 agreed to participate. Three parents declined due to lack of time, and nine parents did not return the questionnaire despite a reminder. All 12 invited nurses participated in the study (Tables 1 and 2). All participants who were approached for the qualitative part, four nurses and eight parents, agreed to participate. The nurses who participated in this part were specialists in Neurology, Nephrology, Muscular diseases, and Pulmonology (Table 1). The children of the participating parents had various diseases (Table 2).

**Table 1:** Baseline characteristics of parents

Characteristics of parents	Quant. (*N* = 22)	Qual. (*N* = 8)
Gender, *N* (%)		
Female	17 (23)	6 (75)
Male	5 (77)	2 (25)
Age, mean (±*SD*)	38 (7.7)	43 (7)
Cultural background, *N* (%)		
Dutch	21 (96)	7 (88)
Not Dutch	1 (4)	1 (12)
Educational level, *N* (%)		
High school	2 (9)	‐
Trade school	9 (41)	2 (25)
Bachelors' degree	7 (32)	3 (38)
Masters' degree	4 (18)	2 (25)
Child age, mean (±*SD*)	8 (6.1)	10 (7.9)
Number of other children, mean (±*SD*)	1.4 (1.2)	1.2 (1)
Child illness (%)		
Autoimmune	1 (5)	‐
Gastroenterology	4 (18)	2 (25)
Neurology	4 (18)	2 (25)
Pulmonology	6 (27)	2 (25)
Diverse syndromes	7 (32)	2 (25)
Duration of child's illness, mean (±*SD*)	6 (5.1)	6.3 (5.4)

**Table 2:** Baseline characteristics of nurses

Characteristics of nurses	Quant. (*N* = 12)	Qual. (*N* = 4)
Gender, female, *N* (%)	12 (100)	4 (100)
Age, mean (±SD)	45 (12.2)	49 (11.6)
Cultural background, Dutch, *N* (%)	12 (100)	4 (100)
Educational level, *N* (%)		
Trade school	3 (25)	2 (50)
Bachelors' degree	6 (50)	1 (25)
Masters' degree	3 (25)	1 (25)
Nursing specialization, *N* (%)		
Nurse specialist	3 (25)	1 (25)
Specialized nurse	9 (75)	3 (75)
Working experience (years), mean (±*SD*)	15 (12.2)	24 (13.4)
Illness specialization, *N* (%)		
Gastroenterology	2 (16)	‐
Muscular diseases	1 (8)	1 (25)
Nephrology	1 (8)	1 (25)
Neurology	1 (8)	1 (25)
Pulmonology	7 (58)	1 (25)

### 3.2 Quantitative part

The relevance was expressed in the CVI (Table 3). The total I‐CVI of the individual items ranged from 0.56 to 1. Items 10, 12, and 24 had a score < 0.78 and were considered to be less relevant by the participants; responses to these items ranged from irrelevant to very relevant. Item 12 was rated low, both by parents (0.52) and by nurses (0.50). The total S‐CVI score was 0.88; 0.80 is considered to be the lower limit of acceptability for an S‐CVI. Parents, on average, rated the relevance slightly lower than nurses, with an S‐CVI of 0.85 and 0.92, respectively.

**Table 3:** Content validity index (I‐CVI nurse, I‐CVI parent, I‐CVI total, S‐CVI)

Item	Expression	I‐CVI parent	I‐CVI nurse	I‐CVI total
1. When problems arise with my child I handle them pretty well	Behaviours	0.96	0.83	0.91
2. I feel confident in my ability to help my child grow and develop	Attitude	0.91	0.92	0.91
3. I know what to do when problems arise with my child	Knowledge	1	1	1
4. I feel my family life is under control	Attitude	0.86^a^	0.92	0.98
5. I am able to get information to help me better understand my child	Knowledge	0.86	1	0.91
6. I believe I can solve problems with my child when they happen	Attitude	0.91	0.83	0.88
7. When I need help with problems in my family I am able to ask for help from others	Knowledge	0.86	0.92	0.88
8. I make efforts to learn new ways to help my child grow and develop	Behaviours	0.77^a^	0.83	0.78
9. When dealing with my child, I focus on the good things as well as the problems	Behaviours	0.81	0.92	0.85
10. When faced with a problem involving my child, I decide what to do and then do it	Behaviours	0.68	0.83	0.74
11. I have a good understanding of my child's disorder	Knowledge	0.90	1	0.94
12. I feel I am a good parent	Attitude	0.52^a^	0.5	0.56
13. I feel that I have a right to approve all services my child receives	Attitude	0.72	0.91	0.79
14. I know the steps to take when I am concerned my child is receiving poor services	Knowledge	0.95^b^	1	0.97
15. I make sure that professionals understand my opinions about what services my child needs.	Behaviours	0.81^a^	1	0.88
16. I am able to make good decisions about what services my child needs	Knowledge	0.96	1	0.97
17. I am able to work with agencies and professionals to decide what services my child needs	Knowledge	0.96	1	0.97
18. I make sure I stay in regular contact with professionals who are providing services to my child.	Behaviours	1	1	1
19. My opinion is just as important as professional's opinions in deciding what services my child needs	Attitude	0.82	0.92	0.85
20. I tell professionals what I think about services being provided to my child	Behaviours	0.89	0.98	0.85
21. I know what services my child needs	Knowledge	0.77	1	0.85
22. When necessary, I take the initiative in looking for services for my child and my family	Behaviours	1	1	1
23. I have a good understanding of the service system that my child is involved in	Knowledge	0.89	0.96	0.85
24. Professionals should ask me what services I want for my child	Attitude	0.68	0.92	0.77
S‐CVI, ≥ 0,9 is good content validity		0.85	0.92	0.88

a 1 missing: n=1

b 2 missing: n=2

### 3.3 Qualitative part

There was no difference between the interpretations of nurses and parents. Therefore, the responses were combined (Table 4).

**Table 4:** Codes of issues with examples

Types of issues	Definitions	Items ≥3 × problems noted	Examples/quotes
Unclear wording	Comments on a word or sentence that can be understood in several ways, multiple meanings	1, 6, 7, 9	Uncertainty about words as “problems” and “pretty well” and problems is a broad concept Mother about Item 7: “Ask for help? Yes, I can. Do I do that? Well no.”
Distinction of items	Comments about items resemble each other	1, 6	No distinction between items
Tone of wording	Comments about wording that is confusing, offensive. Or makes the overall tone of the item overly negative	10, 12, 14, 15, 20, 24	Mother about Item 15: “Well, ‘I make sure’ that is a little firm, something like a fist on the table, now I am really chairing they understand me that they really know what I am thinking.” Mother about Item 24: “Yes, more about tone, I think the question is clear, but I would like to do it together with mutual trust and respect. And I do not want to claim to know everything.”
Perspective	Participants have another perspective on the subject of the item	10, 13, 14, 15, 16, 19, 21, 24	Mother about Item 10: “But ‘I decide what to do and then do it,’ no, it isn't really an item about empowerment because I think it's very stupid if you do that.” Mother about Item 16: “If your child is aware of the problem, I can make good decisions. However, if I am not entirely clear, what kind of problems it is this time and what assistance could fit, I do not make a decision but I first ask for help.”
Feasibility	Comments about wondering if it is feasible what the item poses, participants understand the item	6, 10, 11, 12, 13, 18, 23	Mother about item 13: “Because I think that, you can really put the parent's interest in mind rather than the child's interest.” Item 23: care system is complicated
Reliable answer	Comments about the possibility parents may not give a reliable answer to the item or the item is experienced as subjective	12	Mother about Item 12: “Yes, that's always difficult, it's always so difficult. And it's essential for empowerment to have a real image of yourself.” Father about Item 12: “You should actually ask my son. Yes, I can't quite place this item in the empowerment context.”
Introduction			Several interpretations: independent reply of items, how do parents deal with the illness of their child, does the parent have an opinion in the child's care?
Concept empowerment			Having power, dealing with problems/illnesses, standing up for yourself, managing yourself and problems, organizing everything for your child, can also ask for help in time

Inconsistent interpretations were observed in the first part of the questionnaire, in the domain of family (Items 1, 6, 7, and 9). In Item 1, the term “I handle” was interpreted differently. Interpretations about Item 6 emphasized solving the problem instead of “parents believed,” which is the crux of the item. Both nurses and parents reported difficulties with understanding Items 7 and 9. Item 7 was interpreted two ways: to have a social network or to dare to ask for help. Item 9 was difficult to understand and was interpreted as “when there are problems, also look at the good things.” Detailed information about the interpretation of the items can be retrieved from the corresponding author.

During the analysis of the interviews, six codes for comments were identified: unclear wording, distinction of items, tone of wording, perspective on participation, feasibility of items, and getting reliable answers (Table 4).

Unclear wording and distinction of items was especially seen in the first part of the questionnaire in items with inconsistent interpretations. Items 1 and 6 were seen as items with no distinction. Issues about tone of wording and different perspectives on participation were particularly noted in the second part of the questionnaire, the domain of service systems, and Items 10 and 12. In items that were about decision‐making, opinions, or the participation of parents, all parents and two nurses noticed one or more times in one or more items that they misunderstood the notion of “mutual collaboration with professionals or others” in decision‐making. The tone was sometimes even perceived as offensive and egocentric by five parents and two nurses.

Comments about the possibility of not getting a reliable answer on items were especially made about Item 12 by both nurses and parents. This item was described as difficult to answer, and participants wondered if parents would give a reliable answer on this item. Parents more often made comments about feasibility of the subject of items. For example, this was seen on Item 18 (about the difficulties of making contact with a clinician) and on Item 23 (about the complexity of the health care system).

Participants generally described the questionnaire as not difficult. Although most participants could describe several aspects of empowerment, some, however, had never heard of the concept. Parents especially recognized all of the items and often answered with an example from their own situation. Participants had no important additions to add to the questionnaire. They considered that it would be an improvement in care if parents completed this questionnaire regularly, followed by a conversation with the professional.

### 3.4 Converging both quantitative and qualitative parts

The quantitative and qualitative parts of the study were merged, and recommendations were made for adjustments (Table 5).

**Table 5:** Advice about items

Item	Advice
1. When problems arise with my child I handle them pretty well	Modify: “handle” has several interpretations; therefore, no distinction with Item 3.
2. I feel confident in my ability to help my child grow and develop	Retain: unambiguous interpretation, minimal problems.
3. I know what to do when problems arise with my child	Retain: unambiguous interpretation, minimal problems.
4. I feel my family life is under control	Retain: but attention for the word control can be unclear for degree of control.
5. I am able to get information to help me better understand my child	Retain: clear interpretation, sometimes dependent of the context, is no problem.
6. I believe I can solve problems with my child when they happen	Modify: interpretations about solving the problem, not about believe of the parent “I believe I can,” which is the crux of the item. “Problems” is unclear, but unambiguous interpretations depend of context.
7. When I need help with problems in my family I am able to ask for help from others	Modify: ambiguous interpretation, understanding in two ways.
8. I make efforts to learn new ways to help my child grow and develop	Retain: minimal difference in interpretation. Reliability problems solving by explanation in introduction about empowerment (it is not about right or wrong).
9. When dealing with my child, I focus on the good things as well as the problems	Modify: unclear interpretations, starting point looks like problems.
10. When faced with a problem involving my child, I decide what to do and then do it	Modify: unambiguous interpretations, but problems: Deciding is in consultation with professional, children engage in decisions.
11. I have a good understanding of my child's disorder	Retain: but consider if another word for disorder is needed.
12. I feel I am a good parent	Modify: unambiguous interpretations, but difficult to say it for yourself. Problems: reliability, feasibility.
13. I feel that I have a right to approve all services my child receives	Modify: as far as it is in the interests of the child. Parents don't always have the right (welfare of child).
14. I know the steps to take when I am concerned my child is receiving poor services	Retain: problems with unclear wording, but unambiguous interpretation. Tone of item is rigour but does not exclude conversation.
15. I make sure that professionals understand my opinions about what services my child needs.	Modify: tone of item is offensive. Unambiguous interpretation.
16. I am able to make good decisions about what services my child needs	Modify: collaboration with professional.
17. I am able to work with agencies and professionals to decide what services my child needs	Retain: unambiguous interpretation, several minimal problems.
18. I make sure I stay in regular contact with professionals who are providing services to my child.	Retain: unambiguous interpretation, problems no reason to modify item.
19. My opinion is just as important as professional's opinions in deciding what services my child needs	Retain: unambiguous interpretation, different visions on weight of opinion. Conversation about questionnaire is important.
20. I tell professionals what I think about services being provided to my child	Retain: unambiguous interpretation, tone is offensive, maybe modifying other items on tone can change the experience of tone of the entire questionnaire.
21. I know what services my child needs	Retain: unambiguous interpretation, some problems with vision: collaboration with professional.
22. When necessary, I take the initiative in looking for services for my child and my family	Retain: unambiguous interpretation, focus on family not always clear, but retain.
23. I have a good understanding of the service system that my child is involved in	Retain: unambiguous interpretation, parents experienced difficulties in understanding the care system. Conversation about item is important.
24. Professionals should ask me what services I want for my child	Modify: unambiguous interpretation, problems with tone and perspective on collaboration with professional (together).

Two types of issues (i.e., “getting a reliable answer on items” and comments about the feasibility of an item) were not reasons for modification of the questions. The overall purpose of the questionnaire was to gain insight into empowerment, not to obtain a reliable answer or a high score.

Fourteen items either had a consistent interpretation or no more than one participant provided an interpretation that varied substantially from all of the other participants, with minimal problems noted. Sometimes, participants noted problems with an unclear word, but the interpretation was consistent. The I‐CVI for these items were nearly sufficient, except for Items 8 and 21, which parents rated at 0.77. Parents who rated these items as irrelevant commented on Item 8 about getting a reliable answer and on Item 21 about infeasibility. Therefore, these items were considered to be relevant and comprehensible and were recommended to be retained.

Ten items were recommended for modification. Items 1, 6, 7, and 9 were assessed as relevant, but not easily comprehensible. After modification, these items could be retained. Items 10, 12, 13, and 24 were assessed as irrelevant by parents, and Item 12 was categorized as irrelevant by nurses. However, participants who rated items as irrelevant and were interviewed commented that the items were clear and that they recognized the items. Therefore, these items were considered to be comprehensible as well as relevant, although Item 12 had some specific comments. After modification, these items could be retained. Some items (15 and 16) were interpreted correctly but received many comments about perspective on participation and/or tone, and therefore, they were recommended for modification.

Adjustment of all of the items will mainly consist of choosing other words; reconsidering the English version is recommended. No items were advised to be removed from the FES.

The items that were recommended for modification could be subdivided into different expressions of empowerment: Two items addressed knowledge, four addressed attitude, and four behaviour.

While writing this article, two independent researchers assessed the analysis process and discussed the recommendations with the developers of the questionnaire about final adjustments. Some considerations and decisions were made: Adjustment of the word “problems,” which gave problems in clarity of wording in the first part of the questionnaire, was considered not to be useful because the FES is used in different contexts. Additionally, problems with other perspectives of collaboration were considered not to be a reason for adjustment, because it is just the intention of the FES to get insight into the feelings of parents about their degree of participation.

Some words of the FES were adjusted. For example, the word “I feel” was translated in a different way and the word “services” was explained. A new introduction clearly states the goal of the FES, and a column titled “non‐applicable” is added for when questions do not apply in order to prevent an underestimation of the scores. Originally, participants were advised to answer “never” in this situation. Therefore, fewer adjustments are made as advised in this study.

## 4 DISCUSSION

This study showed that the Dutch FES for parents of children with a chronic condition has sufficient content validity. Although the S‐CVI was 0.88, all items were considered relevant when the quantitative and qualitative data were weighted together. Moreover, the Dutch translation of the FES was found to be comprehensive for the assessment of empowerment. Ten items were advised to be modified. In the domain of “Family,” revisions to the clarity of wording are needed for four items. In the domain of “Services,” six items need to be rephrased in order to improve their tone and perspective.

An important finding of the interviews were comments that were made by all parents and some nurses about their perspectives on mutual collaboration with professionals, which were not addressed by the FES. It might be possible that the Dutch parents in this study in the context of a hospital perceive a degree of empowerment where close collaboration with the professional is still very important for them. Feldman et al. described four approaches of professionals in partnership with patients or families: directing, teaching, collaborating, and supporting. These approaches are the result of a variation in the direction of leadership and in the degree of interaction within a situation (Feldman, Ploof, & Cohen, 1999). For example, “collaboration” assumes leadership of families and a high degree of interaction; “supporting” also assumes family leadership but requires less interaction and a high degree of empowerment. It is possible that the parents in this study require a collaborative approach, where increasing their degree of empowerment can result in a supportive approach.

However, parents in this study with long‐term experience in managing their children's illnesses indicated they felt empowered to make decisions in situations that they were familiar with. However, if new problems arose because of the fluctuating course of the chronic disease, they knew that collaboration with a professional was needed. These parents sometimes preferred the “supportive approach” and sometimes preferred the “collaborative approach,” dependent on the situation of their child. Hence, this may explain that experienced parents who felt empowered also commented about the tone of some items and about the lack of a sense of mutuality in the FES.

When looking at the average scores of the I‐CVIs (items), considerable variation was found, especially in items with a low I‐CVI. The interviews substantiate this outcome. It shows that the use of a mixed‐methods design to judge content validity provided a richer and more in‐depth understanding of the content of the FES. The interviews gave the possibility to gain insight into the underlying thoughts and understanding of the quantitative part (Zhang & Creswell, 2013) Therefore, it was possible to give customized advice on the adjustment of the items.

A limitation of this study may be the fact that it was difficult to include lower educated and non‐native parents for both samples. However, parents of children with different chronic diseases, different genders, ages, education levels, and durations of child's illness agreed to participate. It gives enough variation to conclude that the Dutch FES is valid and useful for parents of children with different chronic conditions.

The attention given to parental empowerment, as described in the introduction, correlates with a focus on the care concept of Family Integrated Care, which places parents at the centre of care and empowers them as primary caregivers. This is a challenge for professionals, who must transition from being a direct caregiver to becoming a mentor and coach (Feldman et al., 1999; Patel, Ballantyne, Bowker, Weightman, & Weightman, 2018). Therefore, more attention should be paid to the importance of parental empowerment in health care and associated concepts such as participation, shared decision‐making, and involvement. Empowering families should be a part of the curricula for health care professionals (Gorter, Visser‐Meily, & Ketelaar, 2010).

In the literature, the FES is applied in research to evaluate interventions (Itzhaky & Schwartz, 2001; Kruijsen‐Terpstra et al., 2016; Martínez et al., 2009). Interestingly, participants of this study regarded the questionnaire as an instrument that could be used to give insight into the personal degree of parental empowerment. Parents could fill in the questionnaire each year and talk with their nurse or clinician about their needs and opportunities to further develop their own empowerment. More research is needed about this application of the FES.

The content validity of the Dutch FES for parents of children with a chronic condition can be considered sufficient. More research is needed about the use of the FES in health care services and the needs of parents to increase their empowerment. The current study demonstrates the utility of the FES for Dutch parents and helps focus future research on the use of the FES in health care.

## FUNDING INFORMATION

None.

## CONFLICT OF INTEREST

None declared.
